# Inhibition of the Nrf2-TrxR Axis Sensitizes the Drug-Resistant Chronic Myelogenous Leukemia Cell Line K562/G01 to Imatinib Treatments

**DOI:** 10.1155/2019/6502793

**Published:** 2019-11-18

**Authors:** Lianrong Xu, Yan Zhao, Fei Pan, Mengxia Zhu, Liqin Yao, Yan Liu, Jiangfang Feng, Jie Xiong, Xiuhua Chen, Fanggang Ren, Yanhong Tan, Hongwei Wang

**Affiliations:** Department of Hematology, 2nd Hospital of Shanxi Medical University, Taiyuan, Shanxi 030001, China

## Abstract

Nuclear factor erythroid 2-related factor 2 (Nrf2) is involved in tumor drug resistance, but its role in imatinib resistance of chronic myeloid leukemia (CML) remains elusive. We aimed to investigate the effects of Nrf2 on drug sensitivity, thioredoxin reductase (TrxR) expression, reactive oxygen species (ROS) production, and apoptosis induction in imatinib-resistant CML K562/G01 cells and explored their potential mechanisms. Stable K562/G01 cells with knockdown of Nrf2 were established by infection of siRNA-expressing lentivirus. The mRNA and protein expression levels of Nrf2 and TrxR were determined by real-time quantitative polymerase chain reaction and western blot, respectively. ROS generation and apoptosis were assayed by flow cytometry, while drug sensitivity was measured by the Cell Counting Kit-8 assay. Imatinib-resistant K562/G01 cells had higher levels of Nrf2 expression than the parental K562 cells at both mRNA and protein levels. Expression levels of Nrf2 and TrxR were positively correlated in K562/G01 cells. Knockdown of Nrf2 in K562/G01 cells enhanced the intracellular ROS level, suppressed cell proliferation, and increased apoptosis in response to imatinib treatments. Nrf2 expression contributes to the imatinib resistance of K562/G01 cells and is positively correlated with TrxR expression. Targeted inhibition of the Nrf2-TrxR axis represents a potential therapeutic approach for imatinib-resistant CML.

## 1. Introduction

Chronic myelogenous leukemia (CML) is characterized by the Philadelphia chromosome (Ph) resulting from reciprocal translocation between chromosome 9 and chromosome 22 [t(9; 22) (q34; q11)], eventually forming the breakpoint cluster region-abelson murine leukemia viral oncogene homolog 1 (BCR-ABL1) oncogene, which encodes a constitutively activated tyrosine kinase [1]. Imatinib mesylate (IM), as the first-generation tyrosine kinase inhibitors (TKIs), targeted represses the tyrosine kinase activity of BCR/ABL fusion protein [2]. Either administered alone or combined with other therapies, it has become one of the first-line drugs for the targeted treatment of CML [3]. However, there are still 15% to 25% of patients having primary or secondary drug resistance due to T315I mutation, clonal evolution, overexpression or hyperactivation of some members of the SRC family of kinases, activation of additional pro-oncogenic pathways, leukemia stem cell intrinsic resistance, and mutations in epigenetic regulators [3–5]. Therefore, it is urgent to explore the solutions for overcoming the imatinib resistance in CML treatments.

Nuclear factor erythroid 2-related factor 2 (Nrf2) can activate the expression of a battery of antioxidant response element-dependent genes, such as thioredoxin reductase (TrxR), to regulate cellular defense against electrophilic and oxidative stress [6, 7]. Overexpressed or hyperactivated Nrf2 can participate in tumorigenesis by helping cells escape from stress or by directly promoting cell survival, proliferation, and even metastasis [8, 9]. Notably, Nrf2 was persistently overexpressed in CML and acute myeloid leukemia (AML) patients [10]. Nrf2 expression was higher in high-risk myelodysplastic syndromes (MDS) patients than that of low-risk patients [11]. In addition, high Nrf2 levels were correlated with poor outcomes in MDS patients [11].

Moreover, Nrf2 plays a vital role in the chemoresistance of tumors to several drugs by some ways, such as protecting cells from the production of ROS or electrophiles, preventing the intracellular accumulation of drugs, and actively inhibiting apoptosis and regulating drug-metabolizing enzymes and efflux transporters [12, 13]. Nrf2 can overcome apoptosis and reduce the susceptibility of AML towards chemotherapeutic agents [14, 15]. High Nrf2 expression is related with chemoresistance to Ara-C, DNR, and ATO in AML cell lines and primary AML cells, and knockdown of Nrf2 can increase AML cells predisposition to chemotherapy drugs [11, 16]. Some studies also explored to reverse the drug resistance of human myelogenous leukemia cells and MDS by using Nrf2 inhibitors [11, 17].

Thioredoxin reductase (TrxR) catalyzes to generate reduced oxidized thioredoxins (Trxs) to regulate diverse cellular redox events during cell proliferation, differentiation, and death [18, 19]. TrxR is often overexpressed in many human cancers and seems to affect the aggressiveness of the tumors [18]. It has been found that the expression of TrxR in doxorubicin-resistant K562 cells is higher than that in the parental sensitive cells and the TrxR inhibitor can reverse doxorubicin resistance [20]. In our previous studies, we found that the Nrf2 mRNA expression was upregulated in the human CML cell line K562 and the bone marrow cells of CML patients, and it was gradually elevated along with the progression of the disease stages. In addition, TrxR was upregulated and appeared as a downstream target gene of Nrf2, suggesting that Nrf2 may be another pathogenesis factor of CML besides Ph chromosome [21, 22]. However, whether TrxR expression is also correlated with Nrf2 expression at both mRNA and protein levels in the imatinib-resistant K562 cells and the potential role of Nrf2 in conferring imatinib resistance to K562 cells have not been extensively elucidated so far.

In the present study, we investigate the effects of Nrf2 knockdown on drug resistance, ROS production, cell proliferation, and apoptosis, as well as the relationship between Nrf2 and TrxR expressions in imatinib-resistant K562/G01 cell line.

## 2. Materials and Methods

### 2.1. Cell Culture

Human CML K562 cell line was purchased from the cell bank of Shanghai Institutes for Biological Sciences, the Chinese Academy of Sciences (Shanghai, China). Imatinib-resistant CML K562/G01 cell line was purchased from the Institute of Hematology, Chinese Academy of Medical Sciences (Tianjin, China). K562 cells and K562/G01 cells were incubated in RPMI l640 medium containing 1% of penicillin and streptomycin and 10% fetal bovine serum (FBS) at 37°C under 5% CO_2_ in saturated humidity.

Imatinib, friendly provided by Novartis AG (Basel, Switzerland), was dissolved into 10 mg/ml stock solution with dimethyl sulfoxide (DMSO) and was diluted with saline water before use. As previously described [23], 2 *μ*M imatinib was added in the culture medium to maintain the resistance of the K562/G0l cells, and the cells were cultured for 2 weeks in the absence of fungicide before experiments. Cell culture medium was changed every 1-2 days.

### 2.2. Establishment of Stable Cells with Knockdown of Nrf2

Four siRNA sequences (Table 1) and one random negative control sequence (antisense strand sequence: 5′-TTCTCCGAACGTGTCACGT-3′) were cloned into the lentiviral vector pGCSIL-GFP with the U6-vshRNA-CMV-GFP frame. In terms of our previous experiment of Nrf2 knockdown in K562 [21], the most effective siRNA sequence (antisense strand sequence: 5′-TTGTGTTTAGTGAAATGCCGG-3′) with a targeting sequence located at 1586 locus of *Nrf2* gene (GenBank accession No. NM_006164.3) was selected in preexperiments (Table 2). Lentiviral particles were produced in K562/G01 cells by transiently cotransfecting the control lentiviral vector (NC-GFP-LV) or Nrf2-knockdown lentiviral vector (Nrf2-RNAi-LV) together with helper plasmids pHelper 1.0 (Gag and Pol) and pHelper 2.0 (VSVG) using house-made transfection reagents from Shanghai Genechem Co., Ltd. (Shanghai, China). The vector constructions, verification by sequencing, virus packaging, and collection of the corresponding viral supernatants were performed by Shanghai Genechem Co., Ltd. (Shanghai, China).

K562/G0l cells in the logarithmic growth phase were inoculated on 24-well plates at a density of 5 × 10^4^/ml and cultured for 24 h until the confluence was around 50%. K562/G0l cells were divided into three groups: experiment group infected with Nrf2-RNAi-LV, control group infected with NC-GFP-LV, and uninfected blank group. Cells were infected by lentivirus with the best multiplicity of infection (MOI = 70) obtained in the preexperiments, and the culture medium was changed after 8 h.

A single green fluorescent protein- (GFP-) marked cell was obtained with the limited dilution method. Briefly, some single clones were identified microscopically after culturing for one week in 96-well plates and were translocated into 24-well plates for expansion. The infection efficiency was detected under a laser scanning confocal microscope (LSCM; Olympus, Tokyo, Japan) and by flow cytometry (FCM). The uninfected cells were used as negative controls for evaluating the infection efficiency. The stably-infected single clones were selected with three rounds of limited dilution.

### 2.3. Real-Time Quantitative Polymerase Chain Reaction (RT-qPCR)

Total RNA was isolated using TRIzol reagent (Invitrogen, Carlsbad, CA) according to the manufacturer's instructions, and RNA quality was confirmed by gel electrophoresis. Total RNA (1 *μ*g each sample) was used to synthesize cDNA utilizing the PrimeScript® RT Master Mix Perfect Real Time Reagent Kit (Takara Bio Inc., Shiga Prefecture, Japan). The cDNA was subjected to RT-qPCR using the SYBR® Premix Ex TaqTM II (Tli RNaseH Plus) Reagent Kit (TaKaRa Bio Inc.) and an AB7500 RT-PCR instrument (Applied Biosystems, Foster City, CA, USA). The PCR reaction protocol consisted of two steps: step 1, initial denaturation for 30 s at 95°C; step 2, denaturation for 5 s at 95°C, annealing and extension for 31 s at 60°C, and fluorescence signal acquisition. The reactions had a total of 40 cycles and ended with a melting curve which consisted of 15 s at 95°C, 1 min at 60°C, 15 s at 95°C, and 15 s at 60°C. PCR primer sequences used were listed in Table 3. PCR primers were synthesized by Sangon Biotech Co. Ltd. (Shanghai, China). The experiments were repeated for 3 times and each sample was run in triplicates. PCR product specificity was confirmed by melting curve analysis. Gene expression levels were normalized to the internal control gene GAPDH and calculated with the 2^−ΔΔCT^ method [24].

### 2.4. Western Blot Assay

Cells were harvested and sonicated in the RIPA (radioimmunoprecipitation assay) buffer for 0.5 h on ice. Then, cell lysates were centrifuged at 12,000 rpm at 4°C for 15 min. After collecting the supernatant, protein concentrations were determined with a BCA Reagent Kit (Thermo Fisher Scientific, Waltham, MA, USA). Proteins (30–50 *µ*g) were separated on 10% sodium dodecyl sulfate-polyacrylamide gel electrophoresis (SDS-PAGE) and transferred onto nitrocellulose (NC) membranes. Membranes were blocked with 5% skim milk for 2 h at room temperature and incubated with primary antibodies against Nrf2 (1 : 200 dilution; Santa Cruz Biotechnology, Dallas, TX, USA), TrxR (1 : 200 dilution; Santa Cruz Biotechnology) and *β*-actin (1 : 200 dilution; Bioss Antibodies, Woburn, MA, USA) overnight at 4°C. After being washed by Tris-buffered saline with 0.5% Tween 20 (TBST), membranes were incubated with secondary antibody-horseradish-peroxidase-labeled goat anti-rabbit IgG (1 : 10,000 dilution; ZSGB-BIO, Beijing, China) for 2 h at room temperature. Proteins of interest were visualized using enhanced chemiluminescence kit (EMD Millipore, Burlington, MA, USA). The band intensities were quantified by densitometry with *β*-actin as an internal control using Quantity One image processing software (Bio-Rad Laboratories, Hercules, CA, USA). Western blots of all the experiments were repeated at least 3 times and one representative blotting result is shown for each experiment.

### 2.5. Reactive Oxygen Species (ROS) Analysis

Rhodamine 123 (Sigma-Aldrich, St. Louis, MO, USA) was used as the ROS trapping agent. Three groups of cells were incubated with 1 M DHR (dihydrorhodamine; Sigma-Aldrich, St. Louis, MO, USA) for 1 h, 6 h, and 24 h, respectively. Then, cells were collected and detected by flow cytometry (EPICS® ALTRA™ Flow Cytometer, Beckman Coulter, Inc., Brea, CA, USA). A total of 1 × 10^4^ living cells were analyzed in each sample. The mean fluorescence intensity (MFI) of rhodamine 123 was calculated to indicate the levels of ROS.

### 2.6. Cell Proliferation Assay

Cells were inoculated on a 96-well plate (1 × 10^4^ cells per well) and cultured for 24 h. Imatinib at the doses of 3 *μ*M, 6 *μ*M, 12 *μ*M, 24 *μ*M, and 48 *μ*M, or 0.1 *μ*M, 0.2 *μ*M, 0.4 *μ*M, 0.8 *μ*M and 1.6 *μ*M, was added to the culture medium of K562/G01 cells or K562 cells, respectively, and cells were further cultured for 72 h. Then, 10 *μ*l Cell Counting Kit-8 (CCK-8; Dojindo Molecular Technologies, Inc., Kumamoto, Japan) was added into each well and cells were incubated for additional 1 h at 37°C. The optical density (OD) was determined at 450 nm with a plate reader.

The cell growth inhibitory ratio was calculated according to the formulas specified below, and the dose-response curve was obtained by plotting the cell growth inhibitory ratio at different concentrations. The drug's half inhibitory concentration (IC_50_) was calculated according to the linear regression equation, and then the drug resistance index (RI) was calculated. The calculations were as follows:

Cell growth inhibiting ratio = 100 − (test OD/nontreated OD) × 100, RI = IC_50_ of drug resistant cell line/IC_50_ of sensitive cell line.

### 2.7. Apoptosis Analysis by Flow Cytometry (FCM)

Cells were diluted and seeded on a 24-well plate (1 × 10^5^ cells per well). After culturing for 12 h, cells were treated with imatinib (at a final concentration of 6 *μ*M or 20 *μ*M) for 28 h. Then, cells were collected to analyze the apoptosis ratio by FCM with an Annexin V-PE/propidium iodide (PI) Apoptosis Detection Kit (Nanjing KeyGen Biotech. Co. Ltd, Nanjing, China) according to the manufacturer's protocol. Briefly, after washing with PBS and the binding buffer for one time each, cells were stained with Annexin V/PI for 20 min at room temperature in dark. After washing with the binding buffer once, the labeled cells were detected immediately by a flow cytometer (FACSCalibur, BD Biosciences, Franklin Lakes, NJ, USA). Data were analyzed by the Kaluza software (Beckman Coulter Inc., Brea, CA, USA). The cells in early stages of apoptosis were Annexin V positive and PI negative, whereas the cells in the late stages of apoptosis were Annexin V and PI double positive.

### 2.8. Statistics

Data were analyzed by SPSS 20.0 (IBM, Armonk, NY, USA). All experiments were conducted at least three times, and data are expressed as means ± standard deviation (SD). The data with two groups of means were compared by *t*-test, while multiple groups of means were compared by one-way analysis of variance (ANOVA). The correlation between Nrf2 and TrxR expressions was analyzed by Pearson's correlation. Statistically significant difference was represented by *p* < 0.05.

## 3. Results

### 3.1. Imatinib-Resistant K562/G01 Cell Line Demonstrates Higher Levels of Nrf2 Expression than the Parental K562 Cell Line

**Figure 1:**
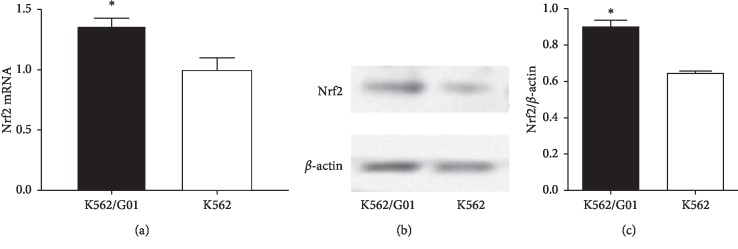
Imatinib-resistant K562/G01 cells had higher levels of Nrf2 expression than the parental K562 cells. (a) The expression levels of Nrf2 mRNA in K562/G01 cells and K562 cells were quantitated by RT-qPCR. (b, c) The expression levels of Nrf2 protein in K562/G01 cells and K562 cells were evaluated by western blot assays. The representative images showed the bands of Nrf2 protein, and *β*-actin was taken as an internal control (b). The relative expression level of Nrf2 protein was quantitated by calculating the densitometry of targeted bands (c). *n* = 3 for each group; ^*∗*^*p* < 0.05, compared between the indicated two cell lines.

In order to determine the role of Nrf2 in imatinib resistance of CML, we first compared the expression levels of Nrf2 in the imatinib-sensitive cell line K562 and the imatinib-resistant cell line K562/G01 by RT-qPCR and western blot assays. As shown in Figure 1(a), the expression level of Nrf2 mRNA was significantly higher (*p*=0.006) in K562/G01 cells (1.37 ± 0.05) than that in K562 cells (1.00 ± 0.08). Consistently, K562/G01 cells also demonstrated significantly higher expression of Nrf2 protein than the parental K562 cells (*p*=0.005) (Figures 1(b) and 1(c)). Therefore, Nrf2 is more expressed in the imatinib-resistant cell line K562/G01 at both transcription and protein levels, which supports our hypothesis that Nrf2 plays a role in imatinib resistance in K562/G01 cells.

### 3.2. Establishment and Verification of Stable GFP-Expressing K562/G01 Cells with Nrf2 Knockdown

**Figure 2:**
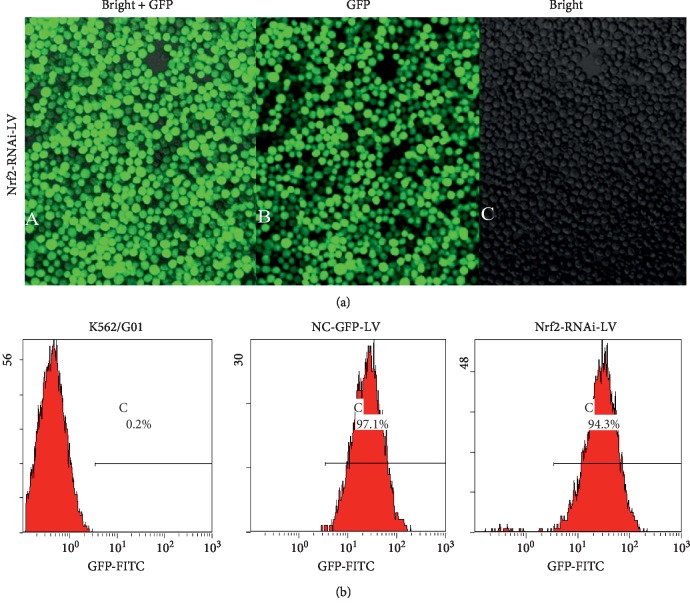
Verification of virus infection efficiency by monitoring GFP expression in K562/G01 cells. (a) Representative images of K562/G01 cells infected with Nrf2-RNAi-LV virus at 488 nm of the nominal optical excitation. Left: merged fluorescent images; middle: FITC fluorescent images; right: transmission photomicrograph at the bright light. Magnification, 10x. (b) Representative histogram plots showed the percentages of GFP-positive cells in uninfected blank K562/G01 cells, NC-GFP-LV-infected cells, and Nrf2-RNAi-LV-infected cells.

We then sought out to establish a stable cell line with Nrf2 knockdown in K562/G01 cells by lentivirus infection. At 72 hours after infection, K562/G01 cells were green fluorescent positive under LSCM, indicating that the viral vector had been successfully delivered into cells. We adopted a limited dilution method to obtain single clones with stable infection. After the stably infected single clones were expanded, almost all the cells showed strong green fluorescence under LSCM (Figure 2(a)). As indicated by the percentages of GFP-positive cells, FCM assay also showed that the infection ratios of NC-GFP-LV control group and Nrf2-RNAi-LV group were (96.1 ± 1.3)% and (93.5 ± 3.8)%, respectively (Figure 2(b)).

### 3.3. Identification of a Positive Correlation between Nrf2 Expression and TrxR Expression in K562/G01 Cells

**Figure 3:**
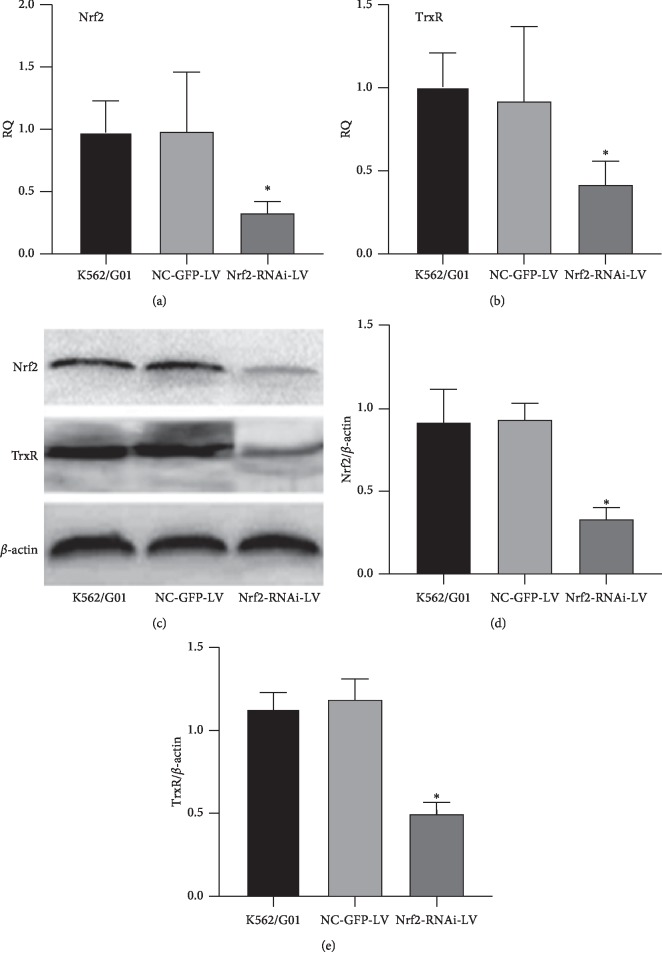
Identification of a positive correlation between Nrf2 expression and TrxR expression in K562/G01 cells. (a, b) The expression levels of Nrf2 mRNA (a) and TrxR mRNA (b) were quantitated in the indicated cells by RT-qPCR. K562/G01, uninfected blank K562/G01 cells; NC-GFP-LV, NC-GFP-LV lentivirus-infected stable K562/G01 cells; Nrf2-RNAi-LV, Nrf2-RNAi-LV lentivirus-infected stable K562/G01 cells. (c–e) The expression levels of Nrf2 protein and TrxR protein were quantitated in the indicated cells by western blot. The representative images showed the bands of targeted proteins, and *β*-actin was taken as an internal control (c). The relative expression levels of Nrf2 protein (d) and TrxR protein (e) were quantitated by calculating the densitometry of targeted bands. *n* = 3 for each group; ^*∗*^*p* < 0.05, compared with the blank K562/G01 cells group.

We verified the knock down efficiency of Nrf2 in the Nrf2-RNAi-LV-infected stable K562/G01 cells by RT-qPCR. As shown in Figure 3(a), the relative expression levels of Nrf2 mRNA was 0.33 ± 0.09, 0.98 ± 0.44, and 0.98 ± 0.21 in Nrf2-RNAi-LV-infected group, NC-GFP-LV control group, and K562/G01 blank group, respectively. Compared with the NC-GFP-LV control group, Nrf2 mRNA was significantly (*p*=0.010) reduced (66.3 ± 0.42)% in the Nrf2-RNAi-LV-infected group. However, no statistical difference (*p*=0.989) on Nrf2 mRNA expression between uninfected K562/G01 cells and NC-GFP-LV control group was observed.

In our previous studies, we found that the TrxR activity of K562 cells was significantly higher than that of normal bone marrow mononuclear cells [19, 20]. After imatinib treatment, the expression levels of TrxR mRNA and protein significantly increased in CML patients than that in the MMR group. In addition, TrxR was overexpressed especially during the progression of CML (AP stage and BC stage) [19, 20]. Therefore, we checked whether TrxR was also expressed more in the imatinib-resistant K562/G01 cells. The expression of TrxR mRNA was 0.42 ± 0.13, 0.92 ± 0.44, and 1.01 ± 0.17 in the Nrf2-RNAi-LV-infected group, NC-GFP-LV control group, and K562/G01 blank group, respectively (Figure 3(b)). Indeed, K562/G01 cells with knockdown of Nrf2 also demonstrate lower expression levels of TrxR mRNA than the other two groups, with a statistical difference (*p*=0.034).

The expression level of Nrf2 protein was decreased ((65.82 ± 2.36)%) in the Nrf2-RNAi-LV-infected group, with a statistical difference (*p*=0.003), compared with that of the NC-GFP-LV control group and K562/G01 blank group. However, no difference (*p*=0.886) between the NC-GFP-LV control group and K562/G01 blank group was observed (Figures 3(c) and 3(d)). These results indicated that Nrf2-RNAi-LV could effectively knock down the expressions of Nrf2 protein in the K562/G01 cells, while NC-GFP-LV had no impact on the expression of Nrf2, which was in line with the RT-qPCR results. Similarly, TrxR protein also demonstrated a significantly lower expression level in the Nrf2-RNAi-LV-infected group than the other two groups, with a statistical difference (*p*=0.001), while no significant difference (*p*=0.933) between the NC-GFP-LV control group and K562/G01 blank group (Figures 3(c) and 3(e)).

Taken together, TrxR showed the same trend of reduced expression as Nrf2 after Nrf2-RNAi-LV infection in K562/G01 cells. Through analysis with Pearson's correlation, it was found that the expression of TrxR mRNA was positively correlated with that of Nrf2 mRNA (*r* = 0.498, *p*=0.036) among the three groups, and this correlation was also present in terms of the expression of TrxR protein among these groups (*r* = 0.998, *p*=0.041) (Table 4).

### 3.4. Knockdown of Nrf2 in K562/G01 Cells Increases the ROS Level

**Figure 4:**
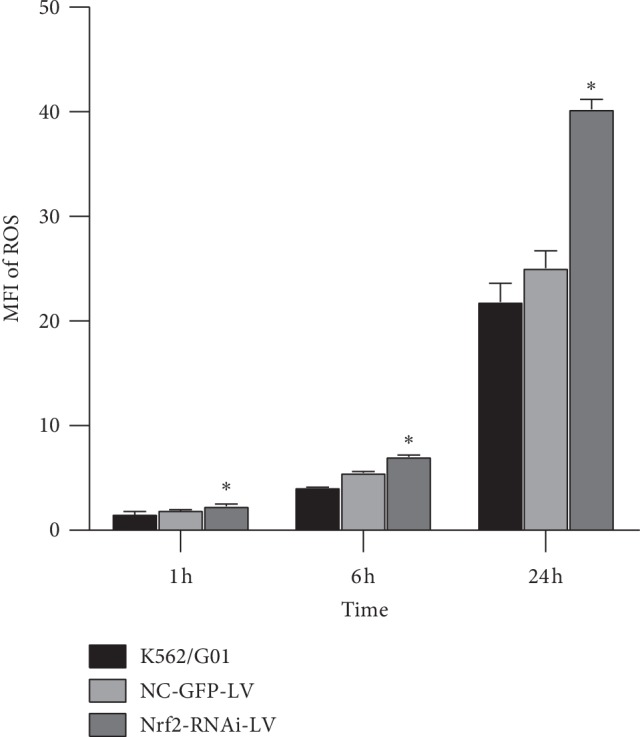
Knockdown of Nrf2 in K562/G01 cells increased the ROS level.

The ROS levels in cell lines of the three groups were detected at three time points of 1 h, 6 h, and 24 h after incubating with dihydrorhodamine, separately. The mean fluorescence intensities of ROS staining in Nrf2-RNAi-LV-infected group were 2.31 ± 0.16, 7.04 ± 0.14, and 40.43 ± 0.78 at 1 h, 6 h, and 24 h, respectively. These values were 1.92 ± 0.05, 5.53 ± 0.10, and 25.20 ± 1.35 in the NC-GFP-LV control group and 1.55 ± 0.21, 4.10 ± 0.05, and 21.95 ± 1.46 in the blank group at 1 h, 6 h, and 24 h, respectively. The Nrf2-RNAi-LV-infected group showed a higher ROS level than that in the other two groups at the same time points with a statistically significant difference (*p*=0.009, 0.001, and 0.001 at 1 h, 6 h, and 24 h, respectively), and no differences between the blank group and NC-GFP-LV control group (*p*=0.056, 0.051, and 0.059 at 1 h, 6 h, and 24 h, respectively) were observed (Figure 4).

The mean fluorescence intensities (MFIs) of ROS staining by Rhodamine 123 in the indicated cell lines were detected after cells were incubated with 1 mol/L DHR for 1 h, 6 h, and 24 h, respectively. *n* = 3 for each group; ^*∗*^*p* < 0.05, compared with the control group and the blank K562/G01 cells group.

### 3.5. Knockdown of Nrf2 Sensitizes K562/G01 Cells to Imatinib Treatments

**Figure 5:**
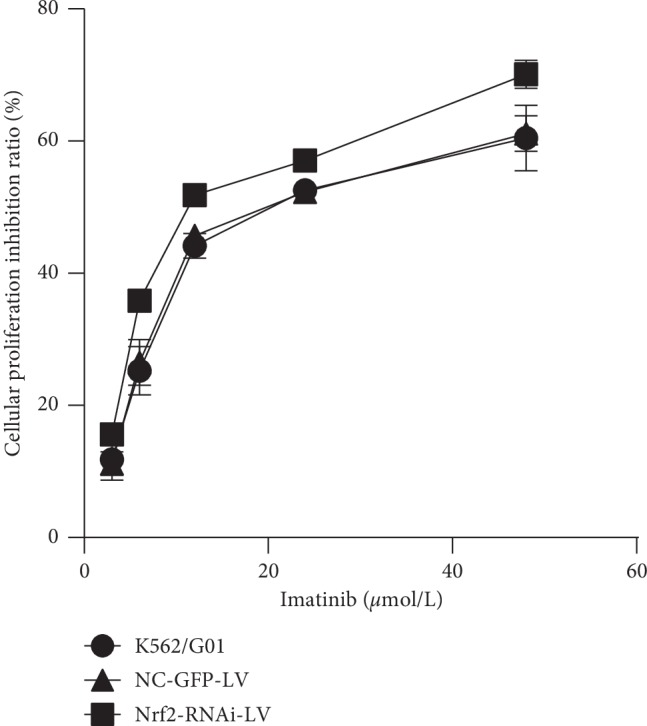
Knockdown of Nrf2-sensitized K562/G01 cells to imatinib treatments.

To substantiate the role of Nrf2 in imatinib resistance, we determined the IC_50_ and RI of parental K562 cells and K562/G01 cells with varied expression levels of Nrf2. Firstly, we identified that the IC_50_ value of K562 cells and K562/G01 cells was 0.663 *μ*mol/L and 22.64 *μ*mol/L, respectively, as measured by the CCK-8 method, and the RI of K562/G01 cells was calculated as 34.28. Knocking down of Nrf2 with siRNA significantly increased the sensitivity of K562/G01 cells to imatinib treatments. The results showed that the IC_50_ of NC-GFP-LV control group and Nrf2-RNAi-LV-infected group was 21.37 *μ*mol/L and 14.64 *μ*mol/L, respectively, and RIs were 32.23 and 22.09, respectively, which suggested that the knockdown of Nrf2 significantly decreased the IC_50_ and RI in K562/G01 cells. As expected, there was no significant difference between the uninfected K562/G01 group and the NC-GFP-LV control group in terms of IC_50_ and RI. The cellular proliferative inhibition ratio in the Nrf2-RNAi-LV-infected group was significantly higher than that in the control group and the blank group at a concentration of imatinib except that with 3 *μ*M (*p*=0.101, 0.010, 0.002, 0.001, and 0.025 at 3 *μ*M, 6 *μ*M, 12 *μ*M, 24 *μ*M, and 48 *μ*M, respectively). However, there was no difference in the control group and the blank group at all concentration of imatinib (*p*=0.755, 0.628, 0.278, 0.558, and 0.826 at 3 *μ*M, 6 *μ*M, 12 *μ*M, 24 *μ*M, and 48 *μ*M, respectively) (Figure 5).

The percentages of cellular proliferation inhibition ratio were calculated in the indicated cell lines after treatments with 3 *μ*M, 6 *μ*M, 12 *μ*M, 24 *μ*M, and 48 *μ*M imatinib for 72 hours. Cell proliferation was accessed with the CCK-8 method. *n* = 3 for each group; ^*∗*^*p* < 0.05, compared with the control group and the blank K562/G01 cells group.

### 3.6. Knockdown of Nrf2 Significantly Increases the Apoptosis Ratio of K562/G01 Cells after Imatinib Treatments

**Figure 6:**
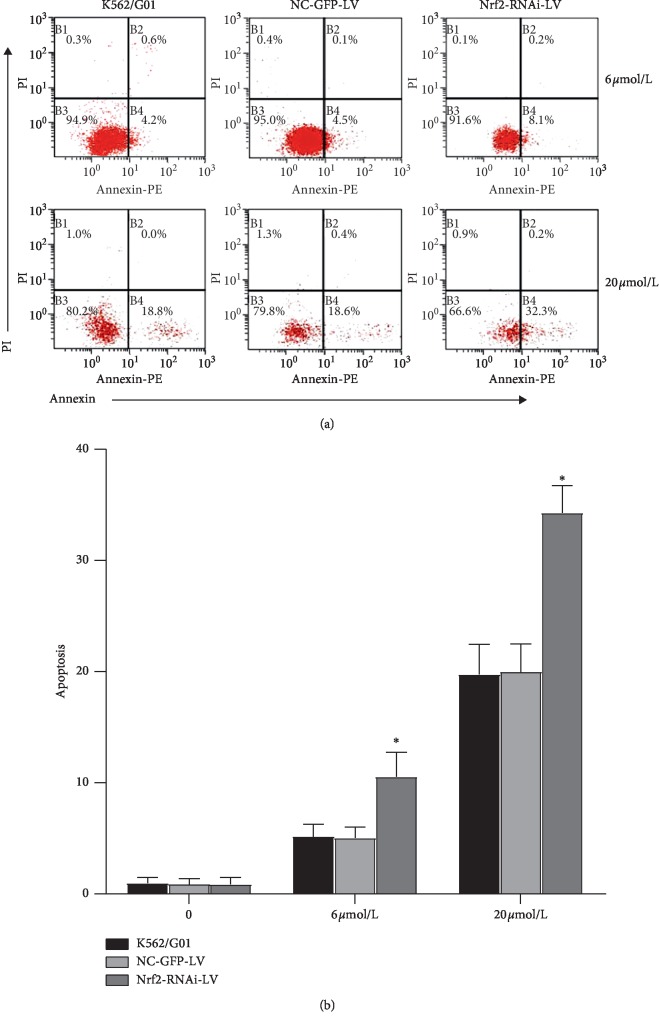
Knockdown of Nrf2 significantly increased the apoptosis ratio of K562/G01 cells after imatinib treatments. (a) Representative flow cytometric profiles showed the Annexin V and PI staining patterns in indicated cell lines at 28 hours after treatments with 6 *μ*M and 20 *μ*M imatinib. (b) Summary data on the percentage of apoptosis ratio (Annexin V-positive cells among total cells) in the indicated cell lines treated with (6 *μ*M and 20 *μ*M) or without (0 *μ*M) imatinib. *n* = 3 for each group; ^*∗*^*p* < 0.05, compared with the control group and the blank K562/G01 cells group.

To further confirm that the K562/G01 cells with Nrf2 knockdown were more sensitive to imatinib treatments, we determined the apoptosis ratio of K562/G01 cells with varied expression levels of Nrf2 after treating with 6 *μ*mol/L or 20 *μ*mol/L imatinib by flow cytometry (Figure 6(a)). Without imatinib treatments, the apoptosis ratios were (0.86 ± 0.51)%, (0.87 ± 0.41)%, and (0.98 ± 0.41)% in the Nrf2-RNAi-LV experimental group, NC-GFP-LV control group, and blank group, respectively. After treating with imatinib at 6 *μ*mol/L and 20 *μ*mol/L, the apoptosis ratios were (10.58 ± 1.82)% and (34.46 ± 1.99)% in the Nrf2-RNAi-LV-infected group, (5.04 ± 0.83)% and (20.09 ± 2.06)% in the NC-GFP-LV control group, and (5.18 ± 0.9)% and (19.86 ± 2.22)% in the blank group, respectively (Figure 6(b)). Apoptosis ratio was significantly higher (*p*=0.007 and 0.001 at 6 *μ*mol/L and 20 *μ*mol/L, respectively) in the Nrf2-RNAi-LV-infected group than that in other two groups regardless of the imatinib concentration, while there was no difference between the apoptosis ratios in blank control K562/G01 cells and NC-GFP-LV control group (*p*=0.916 and 0.917 at 6 *μ*mol/L and 20 *μ*mol/L, respectively) (Figure 6(b)).

## 4. Discussion

It has been reported that Nrf2 inducer can increase resistance to imatinib in K562 cells, suggesting that Nrf2 is involved in imatinib resistance of CML [25, 26]. In the present study, we used the imatinib-resistant BCR/ABL^+^ cell line K562/G01 to investigate the role of Nrf2 in conferring imatinib resistance in CML. We found that K562/G01 cells have higher levels of Nrf2 expression than the parental K562 cells. However, the mechanism that controls Nrf2 expression in drug resistance of CML is presently unknown due to the complex cross-talks between Nrf2 and many other signaling pathways. In addition, the stable K562/G01 cell line with knockdown of Nrf2 was established after siRNA-expressing lentivirus infection of parental cells, and a positive correlation between Nrf2 expression and TrxR expression was observed [21]. Knockdown of Nrf2 in K562/G01 cells increased the ROS level and sensitized the cells to imatinib treatments. We conclude that the expression of Nrf2, in conjunction with TrxR expression, is involved in imatinib resistance of CML and the Nrf2-TrxR axis could be used as a therapy target for imatinib-resistant CML.

The molecular mechanisms for the relationship between TrxR and Nrf2 are likely complex and multifaceted. Over the years, various reports on the relationship between Nrf2 and TrxR have been inconsistent: whether TrxR synergizes with Nrf2 or attenuates the roles of Nrf2 seems to be case-dependent. Cebula et al. summarized that TrxR may be viewed as a potent Nrf2 regulator and gatekeeper of Nrf2 activation [27], whereas someone reported loss of TrxR activity can signal Nrf2 activation without a general oxidative stress. TrxR can be directly inhibited by high ROS levels through an oligomerization process. In multiple myeloma cells, TrxR inhibition induces HO-1 expression through the Nrf2 accumulation transcriptional machinery simultaneously and significantly increased intracellular ROS levels [28]. In our previous studies, we found that despite achieving complete remission in AML or MMR in CML, the expression of TrxR was still higher than normal [22, 29]. After knockdown of Nrf2 in K562 cell line, TrxR was decreased synchronously [21]. In this study, we confirmed that the levels of TrxR mRNA and protein were decreased in concert with Nrf2 in K562/G01 cells infected with Nrf2-RNAi-LV lentivirus. Therefore, it appeared that TrxR, as one of Nrf2 target genes, was coordinated with Nrf2 to be involved in the drug resistance of CML. It is worth noting that the TrxR system not only controls intracellular ROS levels and redox events but also itself is regulated by redox processes, leading to the potential for autoregulatory loops. Therefore, the mechanisms between Nrf2 and TrxR in CML and imatinib-resistant CML are likely to be complex, and the effects of various factors may be different depending on the cell environment and redox state.

ROS and oxidative stress have long been associated with cancer [30, 31]. Accumulating evidence supports that ROS are bifaceted in cellular processes. Modest levels of ROS are required for cancer cells to survive, whereas excessive levels destroy them [30, 31]. In our study, the intracellular ROS significantly increased in a time-dependent mode in infected K562/G01 cell line with Nrf2 knockdown and TrxR decreasing, although ROS also increased with time in the uninfected group attributed to BCR-ABL constitutively producing intracellular ROS [32, 33]. It is reported that increased activity of antioxidant genes by Nrf2 in cancer cells can repress p53-dependent apoptosis; the latter requires the accumulation of ROS, oppositely, the loss of Nrf2 increases ROS [34]. In multiple myeloma, intracellular ROS levels are increased when TrxR is restrained [35]. Nrf2 may also regulate sensitivity to ROS-producing therapeutic agents [6]. Therefore, we speculated that the downregulation of Nrf2 and TrxR may lead to inhibition of their biological activity, compromised ability as the cellular antioxidant or cause increased level of intracellular ROS to promote the apoptosis of K562/G01. However, whether this elevated ROS can continue to eradicate CML cells is warranted. Moreover, how to define and weigh the effect of the production of ROS on imatinib resistance of CML is challenging.

The IC_50_ value of imatinib for K562/G01 cells was about 31.14-fold higher than that of K562, suggesting that K562/G01 cells had acquired significant resistance to imatinib. Knocking down of Nrf2 increased the sensitivity of K562/G01 cells to imatinib and the drug resistance index decreased. The K562/G01 cell line is an imatinib-resistant cell line established by inducing the K562 cell line by low doses of imatinib, but it has no BCR/ABL gene mutation [23]. Therefore, the present results indicated that Nrf2 was involved in imatinib resistance of K562/G01 with independence of BCR-ABL. Other studies also have found that Nrf2 knockdown in the resistant cells can increase the sensitivity of tumor cells to chemotherapeutic agents. For example, the silencing Nrf2 can increase the sensitivity of cisplatin-resistant ovarian cancer cell strain to cisplatin and that of lung cancer cells to doxorubicin [36, 37]. Similar reports also presented in studies involving gallbladder cancer and colon cancer [38, 39].

In the present study, the apoptosis ratios were similarly low in both the infected and noninfected groups when Nrf2 was knockdown. Apoptosis was significantly increased in the infected group in a dose-dependent manner after cells were treated with imatinib. Recent studies have demonstrated that Nrf2 acts as a dual role in cancers: it protects the survival of benign from chemical carcinogenesis and environmental stresses, whereas it provides advantages for the development of cancer cells [40]. Probably, this is the reason for no significant difference in apoptosis rates between the infected and the noninfected group. In the infected group, the increase in apoptosis rate was mainly caused by the addition of imatinib, suggesting that the sensitivity of the infected group to imatinib was restored after Nrf2 gene knockdown. Collectively, our data suggest that Nrf2 expression confers imatinib resistance to K562/G01 cells.

## 5. Conclusions

In summary, our *in vitro* preliminary study confirms that Nrf2 expression is a key factor of resistance to imatinib in K562/G01 cell lineage with independence of BCR-ABL mutation. Downregulating Nrf2 expression in K562/G01 cells was companied with TrxR decreasing, which promoted imatinib-induced apoptosis, suppressed cell proliferation, and enhanced the intracellular ROS level to activate oxidation stress system in K562/G01 cells. Although in lack of *in vivo* animal studies and clinical data, our work indicates that the Nrf2-TrxR axis is a potential target for reversing the drug resistance in CML. How to smoothly transfer its functions between normal cells and tumor cells to achieve the cytoprotection of normal cells and maintaining of anti-tumor effects is more complex. Therefore, it will be necessary to understand the molecular regulations of Nrf2/TrxR and identify the individualized status of Nrf2/TrxR expression in imatinib-resistant CML.

## Figures and Tables

**Table 1:** Four siRNA sequences.

No. 1 forward strand	GCAGCAAACAAGAGATGGCAA
No. 1 reverse strand	TTGCCATCTCTTGTTTGCTGC
No. 2 forward strand	GCACCTTATATCTCGAAGTTT
No. 2 reverse strand	AAACTTCGAGATATAAGGTGC
No. 3 forward strand	CCGGCATTTCACTAAACACAA
No. 3 reverse strand	TTGTGTTTAGTGAAATGCCGG
No. 4 forward strand	CCCTGTTGATTTAGACGGTAT
No. 4 reverse strand	ATACCGTCTAAATCAACAGGG

*Note*. No. 3 is the target sequence (from 5′ to 3′).

**Table 2:** Target and control sequences established.

	Sequence
Frame structure	U6-vshRNA-CMV-GFP

A framework to be established	5′-CCGG + sense strand + loop CTCGAG + antisense strand + TTTTTG-3′
	5′-AATTCAAAAA + sense strand + loop CTCGAG + antisense strand-3′

Targeted sequence (Nrf2-RNAi-LV)	Sense strand siRNA: CCGGCATTTCACTAAACACAA
	Antisense strand siRNA: TTGTGTTTAGTGAAATGCCGG

Control sequence (NC-GFP-LV)	Target sequence: TTCTCCGAACGTGTCACGT

**Table 3:** Primer sequences of each gene used for RT-qPCR (from 5′ to 3′).

Gene	GenBank serial number		Primer sequences	Product (bp)
Nrf2	NM_006164.3	Forward	ACAATGAGGTTTCTTCGGCTAC	141
		Reverse	CGTCTAAATCAACAGGGGCTAC	

TrxR	NM_003330.2	Forward	TATCAGGAGGGCAGACTTCAA	153
		Reverse	GACCATCACCTTCTTGCCATA	

GAPDH	BC004109	Forward	AGAAGGCTGGGGCTCATTTG	258
		Reverse	AGGGGCCATCCACAGTCTTC	

**Table 4:** The impacts of siRNA-mediated Nrf2 knockdown to the mRNA expressions of Nrf2 and TrxR in K562/G01 cells.

Gene	Group	ΔCT ($\bar{x}\pm s$)	RQ ($\bar{x}\pm s$)
Nrf2	K562/G01	3.15 ± 0.30	0.98 ± 0.21
	NC-GFP-LV	3.23 ± 0.90	0.98 ± 0.44
	Nrf2-RNAi-LV	4.63 ± 0.29	0.33 ± 0.09^*∗*^

TrxR	K562/G01	19.22 ± 0.25	1.01 ± 0.17
	NC-GFP-LV	18.97 ± 0.68	0.92 ± 0.44
	Nrf2-RNAi-LV	20.50 ± 0.31	0.42 ± 0.13^*∗*^

^*∗*^ *p* < 0.05, compared with the uninfected K562/G01 group or NC-GFP-LV-infected group; *n* = 3 for each group.

## Data Availability

The data set supporting the results of this article are included within the article.
